# IL-22 Confers EGFR-TKI Resistance in NSCLC via the AKT and ERK Signaling Pathways

**DOI:** 10.3389/fonc.2019.01167

**Published:** 2019-11-05

**Authors:** Xiaomeng Wang, Jiali Xu, Jin Chen, Shidai Jin, Jiaqi Yao, Tongfu Yu, Wei Wang, Renhua Guo

**Affiliations:** ^1^Department of Oncology, The First Affiliated Hospital of Nanjing Medical University, Nanjing, China; ^2^Department of Radiotherapy, II, The First People's Hospital of Shangqiu, Shangqiu, China; ^3^The Fourth Clinical Medical College, Nanjing Medical Universtiy, Nanjing, China; ^4^The First Clinical Medical College, Nanjing Medical Universtiy, Nanjing, China; ^5^Department of Radiology, The First Affiliated Hospital of Nanjing Medical University, Nanjing, China; ^6^Department of Cardiothoracic Surgery, The First Affiliated Hospital of Nanjing Medical University, Nanjing, China

**Keywords:** IL-22, non-small cell lung cancer, EGFR, gefitinib, EGFR-TKI resistance

## Abstract

**Background:** The efficacy of an EGFR-targeted treatment strategy for non-small cell lung cancer (NSCLC) is reduced by drug resistance. IL-22 enhances tumor growth and induces chemotherapy resistance in human lung cancer cells. The present study elucidated the IL-22-induced mechanism underlying EGFR-tyrosine kinase inhibitor (TKI) resistance in NSCLC.

**Methods:** The plasma and tissues of patients who received EGFR-TKIs were utilized to determine the association between IL-22 expression and gefitinib efficacy. The IL-22 effect on the EGFR/ERK/AKT pathways in NSCLC HCC827 and PC-9 cells was determined using the CCK-8 assay, western blot, and flow cytometric analysis. A PC-9 xenograft model of IL-22 exposure was established. Gefitinib was administered to mice in combination with IL-22 or vehicle.

**Results:** We showed that IL-22 expression was higher in the EGFR-TKI-resistant group compared to EGFR-TKI-sensitive group. IL-22 expression was associated with EGFR-TKI efficacy in plasma. Additional treatment of IL-22 induced gefitinib resistance and reduced apoptosis in PC-9 and HCC827 cell lines. Furthermore, we showed that the effects of IL-22 attributed to p-ERK, p-EGFR, and p-AKT up-regulation. IL-22 neutralizing antibody completely abrogated the effects of IL-22 on apoptosis and AKT/EGFR/ERK signaling. Finally, we showed that IL-22 enhanced tumor growth and induced gefitinib resistance in the PC-9 xenograft model. Moreover, compared with gefitinib alone, the combination of IL-22 and gefitinib led to an increase in Ki67-positive staining and a reduction in TUNEL staining.

**Conclusions:** Our findings indicate that IL-22 plays a role in tumor progression and EGFR-TKI resistance in NSCLC. Thus, IL-22 might serve as a novel biomarker to overcome resistance of EGFR-TKI.

## Introduction

Lung cancer is a main cause of cancer mortality globally, and is responsible for 1.6 million deaths every year (1). Non-small cell lung cancer (NSCLC) represents ~85% of all lung cancers (2). For NSCLC patients with EGFR-activating mutations, EGFR tyrosine kinase inhibitors (EGFR-TKIs), such as erlotinib, gefitinib and dacomitinib, have dramatic therapeutic effects. Based on the promising results of several clinical trials, the National Comprehensive Cancer Network (NCCN) has recommended EGFR-TKIs as standard therapy for advanced NSCLC patients with EGFR sensitive mutations (3–6). However, acquired resistance invariably develops ~10 months after therapy (7). Secondary-site EGFR mutations, most of which are T790M, are the major cause of acquired resistance (~60%). Other mechanisms of acquired resistance include PIK3CA mutation (<5%) (7, 8) and MET gene amplification (5–10%). Osimertinib is a third-generation EGFR-TKI developed for the EGFR-TKI resistance with T790M and has demonstrated great efficiency. Furthermore, immune checkpoint (programmed death 1 [PD-1] and programmed death-ligand 1 [PD-L1]) inhibitors have been tried for these patients, although no satisfactory efficacy was achieved so far. Despite these efforts, the mechanisms of EGFR-TKI resistance are not fully understood. Also, there is enormous need to develop innovative treatment strategies based on the mechanism underlying EGFR-TKI resistance.

Former studies have shown that besides tumor cells, the microenvironment of tumor also contributes to drug resistance (9, 10). The tumor microenvironment is a complex system containing a variety of inflammatory cells and mediators. Tumor-associated macrophages (TAMs) are the most abundant inflammatory cells in tumor microenvironment (11–13). TAMs can promote tumor cell proliferation, metastasis, and mediate acquired resistance to chemotherapy by releasing inflammatory factors such like interleukin-6 (IL-6), TGF-β, IL-10, and TNF-α (14–21).

IL-22 is an IL-10 cytokine, which is mainly produced by immune cells such like NK cells, T-helper (Th) cells, and innate lymphocytes (22–24). Neither resting nor activated immune cells express IL-22 receptors (25). IL-22 represents a late-model of immune mediator that, produced by immune cells, regulates tissue cells of the digestive tract, skin, lungs, and kidney (26). Elevated levels of IL-22 are relevant to a good amount of tumors, such as gastric, lung, pancreatic, colon, and liver cancers (27). Recent evidences suggested that many cancer cell lines, including those originating from the lung, express the interleukin-22-receptor 1 (IL-22-R1) (28). Zhang et al. (29) proposed that IL-22 is an essential mediator during the activity between lung cancer cells and the immune environment. Moreover, up-regulation of IL-22 promotes tumor proliferation via the AKT signaling pathway (30) and induces chemotherapy resistance in human lung cancers (31). However, the role of IL-22 in inducing EGFR-TKI resistance is unknown.

To explore if IL-22 plays a role in EGFR-TKI resistance, we measured the IL-22 expression in the tissues and the dynamic changes in plasma of patients receiving gefitinib as first-line therapy. *In vivo* and *in vitro* experiments were also performed to explore the potential signaling pathway involved. Our data suggests that IL-22 plays an important role in EGFR-TKI resistance and may serve as a therapeutic target.

## Materials and Methods

### Ethical Statement

This study was carried out in accordance with the recommendations of institutional guidelines and Local Ethics Committee of the First Affiliated Hospital of Nanjing Medical University. All patients gave written informed consent in accordance with the Declaration of Helsinki. The protocols including animal experiment were approved by the Local Ethics Committee of the First Affiliated Hospital of Nanjing Medical University. The institutional guidelines of the Animal Care and Use of Nanjing Medical University were followed for the welfare of the animals.

### Tissue and Plasma Samples

Twenty advanced lung adenocarcinoma tissue samples and paired post- and pre-treatment samples of plasma were obtained from NSCLC patients. Of the 20 tissue samples, 10 were obtained from patients after the development of acquired resistance regarding EGFR-TKI. The other 10 samples were obtained from patients sensitive to EGFR-TKI therapy. Five paired plasma samples were obtained from patients at three time points: before EGFR-TKI therapy (pre-treatment); while sensitive to EGFR-TKI therapy (post-S); and when resistance was acquired to EGFR-TKI therapy (post-R). All of the samples had EGFR mutations involving an exon 19 deletion (19DEL) or exon 21 mutation (L858R) among patients who were treated with an EGFR-TKI (gefitinib or erlotinib) from March 2015 to August 2017.

### Reagents and Cell Culture

The EGFR del E746-A750 mutated cell lines of human lung adenocarcinoma (HCC827 and PC-9) were used. The PC-9 cell line was provided by Professor Zhou Caicun of the Department of Oncology at Shanghai Pulmonary Hospital. HCC827 was kindly provided by the Cell Bank of the Chinese Academy of Sciences. We cultivated cells in RPMI-1640 medium (Gibco, Carlsbad, CA, USA), which was supplemented with FBS of 10% at 37°C in CO_2_ of 5%. IL-22, human IL-22 monoclonal antibody (MAB7821), and mouse IgG_1_ isotype control (MAB002) were obtained from R&D Systems (Minneapolis, MN, USA). Gefitinib was obtained from Selleckchem (Houston, TX, USA).

### Reverse Transcription, Quantitative Real-Time Polymerase Chain Reaction, and RNA Extraction

We extracted total RNA from tissues using Trizol reagent (TaKaRa, Tokyo, Japan). We synthesized cDNA using Primescript RT (TaKaRa) per the instructions of the manufacturer. We used the following PCR primers: 5′−*GCTTGACAAGTCCAACTTCCA*−3′ (IL-22 forward); 5′−*GCTCACTCATACTGACTCCGT*−3′ (IL-22 reverse); 5′−*GCTGTGCTATCCCTGTACGC*−3′ (β-actin forward); and 5′−*TGCCTCAGGGCAGCGGAACC*−3′ (β-actin reverse). A quantitative real-time polymerase chain reaction for IL-22 and β-actin was performed for each sample of cDNA using FastStart Universal SYBR Green Master (Rox) following the instructions of the manufacturer. The gene expression was normalized and β-actin served as an internal control. We performed the experiments in triplicate. The median value was used to compute the relative IL-22 concentrations using the following formula: Ct _cycle threshold_ = Ct _median IL-22_ − Ct _medianβ*-actin*_. The 2^−ΔΔCt^ Method was used to compare the relative expression.

### Enzyme-Linked Immunosorbent Assay (ELISA)

IL-22 plasma levels were assessed by ELISA using the human IL-22 ELISA kit (R&D Systems) according to the instructions of the manufacturer. The mean minimum detectable level was 2.7 × 10^−3^ ng/ml. The average storage time of the plasma was 16.5 months (range, 3–30 months). No sample was thawed before testing.

### Cell Counting Kit-8 (CCK-8)

Cell proliferation was assessed using CCK-8 (MedChemExpress, Monmouth Junction, NJ, USA) following the instructions of the manufacturer. We seeded HCC827 and PC-9 cells at a density 3,000 cells/well in a plate with 96 wells in complete medium overnight. Afterwards, the cells were treated with different concentrations (0.01–2 μM) of gefitinib in the presence or absence of IL-22 (50 ng/ml) and incubated the cells in 5% CO_2_ at 37°C for 48 h. Ten microliters of CCK-8 reagent was added to each well, and the absorbance at 450 nm in 1 h was measured. The values were corrected by subtracting the absorbance of control wells that did not contain cells. Cell viability was counted based on the following equation: [(OD control-OD sample)/(OD control- OD blank)] × 100%. The concentration of drug that inhibited the growth of the tumor cells by 50% (IC50) was used to evaluate the drug effect.

### Flow Cytometric Analysis

PC9 and HCC827 cells were seeded on plates with six wells (2 × 10^4^ cells per well), and treated with gefitinib (IC50), IL-22 neutralizing antibody (10^4^ ng/ml), or IL-22 (50 ng/ml) for 48 h. The apoptotic rates of death were obtained using an apoptosis kit based on AnnexinV/PI (EMD Millipore, Billerica, MA, USA) according to the instructions of the manufacturer.

### Western Blot

PC9 and HCC827 cells were lysed by RIPA protein extraction reagent, which was supplied with phenylmethanesulfonyl fluoride. Protein was quantified using the BCA assay (Thermo Scientific, Rockford, IL, USA). In brief, the protein lysates were isolated on 10% sodium dodecyl sulfate polyacrylamide gels, which were electrotransferred to polyvinylidene fluoride membranes. We blocked the transferred membranes with 5% non-fat milk, which were incubated with primary antibodies overnight at 4°C. We incubated membranes with horseradish peroxidase (HRP)-conjugated secondary antibodies (Cell Signaling Technology, Beverly, MA, USA), and visualized the membranes through a chemiluminescent HRP substrate (Millipore Corporation, Billerica, MA, USA). Primary antibodies against EGFR, AKT, ERK, phospho-EGFR, phospho-AKT, and phospho-ERK were purchased from Cell Signaling Technology. The protein levels were normalized to GAPDH (Cell Signaling Technology).

### Tumor Xenografts in Nude Mice

BALB/C female nude mice, 4–6 weeks old and weighing 18–22 g, were purchased from the Research Center of Model Animal (Nanjing University, Nanjing, China). The Research Committee approved this research protocol. Mice were maintained following the institutional ethics guidelines for animal experiments. Mice received a subcutaneous injection with 5 × 10^6^ PC-9 cells. The mice were randomly divided into four groups during the indicated period: treated with vehicle; IL-22 (s.c.); gefitinib (oral); or IL-22 (s.c.) combined with gefitinib (oral). IL-22 (s.c.) and gefitinib (oral) were administered 5 days each week at a dose of 50 μg/kg and 20 mg/kg in 0.5% Tween 80 (Sigma, St. Louis, MO, USA), respectively.

The size of the tumor and weight of the mouse were measured every 3 days. The volume of tumor was defined by the equation, V = ab^2^/2, where “a” was tumor length and “b” was tumor width. The percent tumor growth inhibition (TGI) in each group was computed by the following equation: (TuG_control_-TuG_test_)/TuG_control_^*^100%, where TuG = (final tumor size—pre-treatment tumor size). After 27 days, the mice were sacrificed and paraffin-embedded tissues were obtained for immunohistochemical (IHC) staining.

### Histology and IHC Staining

Tissues were fixed in formaldehyde (v/v) of 10% in PBS. Then, the tissues were embedded in paraffin and cut into 5-μm sections. Tumor tissue sections were deparaffinized in xylene solution and rehydrated. Immunostaining was conducted using an IHC kit (Abcam, San Francisco, CA, USA). Rabbit polyclonal antibodies to EGFR (1:100), ERK (1:200), p-EGFR [pY1068] (1:300), p-AKT [pS473] (1:200), AKT (1:2,000), and p-ERK [Thr202/Tyr204] (1:400) were used as primary antibodies. Ki67 (Cell Signaling Technology) was utilized for the antigen retrieval process, which was conducted in 10 mM citric buffer (pH 6.0) for 20 min in a cooker before staining. The tissues were counterstained with hematoxylin. Apoptotic cells in tumor tissues were evaluated via terminal deoxynucleotidyl transferase (TdT)-mediated dUTP–biotin nick end labeling (TUNEL) stain using an *In Situ* Cell Death Detection Kit (Roche, Mannheim, Germany) according to the manufacturer's protocol. Ki-67 positive cells showed brown granules in the nucleus with or without mild cytoplasmic staining. The positive cells were counted under microscope. Five high power visual fields (×400) were randomly selected for each slice. In each field, 100 cancer cells were counted and the percentage of positive cells was caculated. For the TUNEL assay, five high power visual fields (×400) were randomly selected for each slice. The number of apoptotic cells and total cells were counted. The apoptotic rate was the percent of apoptotic cells to total cells.

### Statistical Analyses

We used SPSS® software (version 24.0; SPSS, Inc., Chicago, IL, USA) to conduct statistics analysis. Data are denoted by the mean ±SEM of triple independent assays. Discrepancies between the groups were assessed using one-way ANOVA analysis and a two-tailed *t*-test. A *P* value < 0.05 was considered as statistically significant.

## Results

### IL-22 Expression Was Associated With EGFR-TKI Acquired Resistance in Human Lung Cancer Tissues and Plasma

**Figure 1:**
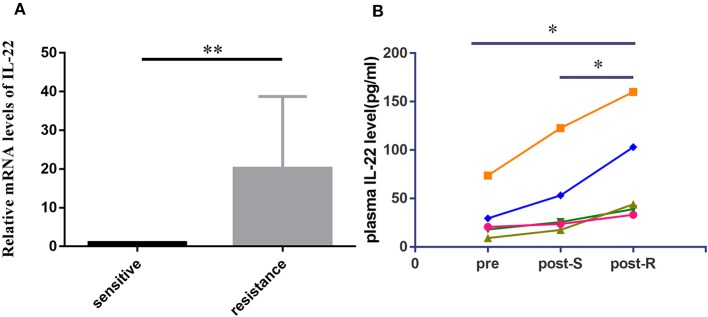
IL-22 expression levels in human lung cancer tissue and plasma. **(A)** IL-22 mRNA expression levels assessed in 20 cases of EGFR-mutant patients before treatment (10 cases) and upon acquired resistance to EGFR-TKIs (10 cases). Data were means of two separate experiments mean ± SEM. **(B)** Pre to post changes in 5 pair of plasma measured with the ELISA assay. Individual patients are depicted with a straight line connecting pre, post-S and post-R. ^*^P < 0.05, ^**^P < 0.01. pre, pre-treatment; post-S, post-treatment (sensitive to EGFR-TKIs); post-R, post-treatment (acquired resistance to EGFR-TKIs).

To detect the expression of IL-22 in lung cancer tissues, we used qRT-PCR to analyze IL-22 mRNA in 20 EGFR-mutant NSCLC patients. The IL-22 mRNA level was lower in the EGFR-TKI-sensitive group than the acquired resistance group (*P* < 0.01; Figure 1A). Paired pre-treatment, post-S, and post-R plasma samples were obtained from five patients for ELISA analysis. IL-22 overexpression was consistently found in post-R plasma compared with pre-treatment (*P* = 0.035) and post-S plasma (*P* = 0.021; Figure 1B).

### IL-22 Plus Gefitinib Induced Acquired Gefitinib Resistance in PC-9 and HCC827 Cells

**Figure 2:**
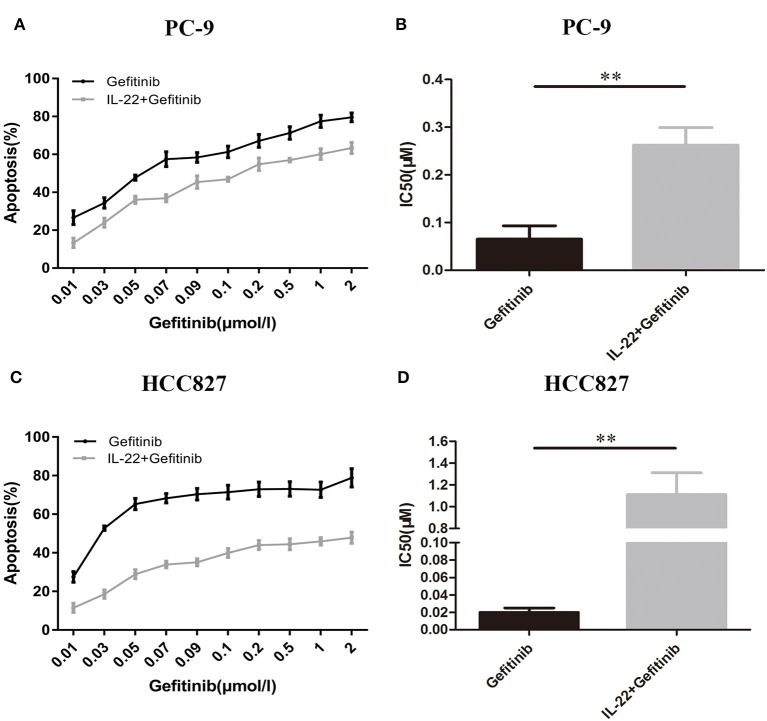
IL-22 induced resistance to gefitinib in PC9 and HCC827 cells *in vitro*. **(A)** PC-9 and **(C)** HCC827 cells were co-treated with gefitinib (0.01–2 μM) with or without IL-22 (50 ng/ml) for 48 h. Cell survival was measured using CCK-8 assay, and was expressed as a percentage of cell viability relative to control cells. **(B)** PC-9 and **(D)** HCC827 cells IC50 values of gefitinib were calculated for each cell. ^**^P < 0.01 for gefitinib plus IL-22 compared with gefitinib alone. Results were presented as the means ± SEM of three independent experiments.

PC-9 and HCC827 cell growth was determined using the CCK-8 assay. As expected, gefitinib inhibited PC-9 growth (Figure 2A), as well as HCC827 growth (Figure 2C), depending on the concentration with IC50 values of 0.065 ± 0.016 μM (Figure 2B) and 0.02 ± 0.0029 μM (Figure 2D), respectively. If cells were treated with gefitinib plus IL-22 (50 ng/ml [48 h]), IC50 values increased and reached 0.26 ± 0.021 μM (PC-9) and 1.11 ± 0.12 μM (HCC827), respectively (Figures 2B,D). These results showed that IL-22 induced resistance of gefitinib in HCC827 and PC-9 cells (*P* = 0.002 and 0.001, respectively).

### IL-22 Protected HCC827 and PC-9 Cells From Apoptosis Induced by Gefitinib

**Figure 3:**
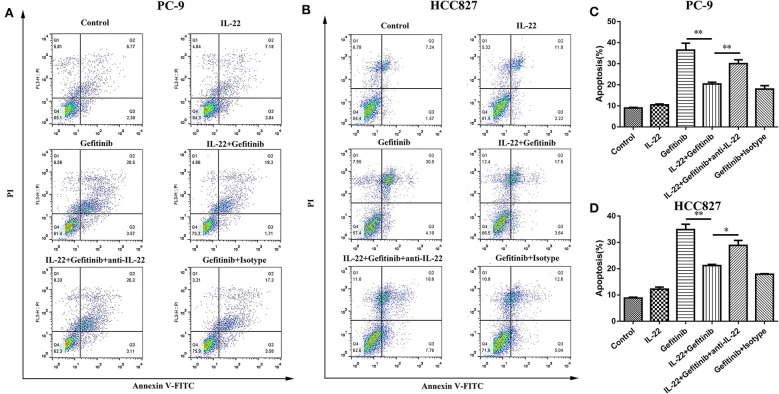
IL-22 protected PC-9 and HCC827 cells from apoptotic death after gefitinib treatment. **(A,B)** Analysis of apoptosis following 6 independent treatments. PC-9 **(A)** and HCC827 **(B)** cells were treated in the presence or absence of gefitinib (IC50) for 48 h prior to exposure to 50 ng/ml IL-22, 10^4^ ng/mL IL-22 neutralizing antibody and mouse IgG_1_ Isotype control for additional 24 h and subjected into Annexin V based-flow cytometric analysis according to the manufacturer's instruction. **(C,D)** The % of apoptotic cell is indicated in right. ^*^P < 0.05, ^**^P < 0.01. anti-IL-22, IL-22 neutralizing antibody; Isotype, mouse IgG_1_ Isotype control.

Because refractoriness to apoptosis is a major feature of EGFR-TKI targeted therapy resistance in NSCLC, the IL-22 effects on cell apoptosis were explored. A lower apoptotic cell percentage was demonstrated in PC-9 cells treated with gefitinib plus IL-22 compared to cells treated with gefitinib alone (20.41% vs. 36.47%, *P* = 0.009; Figures 3A,C). The same effect was observed in HCC827 cells (IL-22 and gefitinib group [21.16%] vs. gefitinib group [34.86%]; *P* = 0.003; Figures 3B,D). The treatment of IL-22 neutralizing antibody could reverse the apoptosis induced by IL-22 combined with gefitinib in PC-9 (*P* value < 0.05) and HCC827 cells (*P* value < 0.01; Figure 3). All the results suggested that IL-22 could protect HCC827 and PC-9 cells from apoptosis induced by gefitinib.

### IL-22 Promoted Phosphorylated EGFR/AKT/ERK Activation in HCC827 and PC-9 Cells

**Figure 4:**
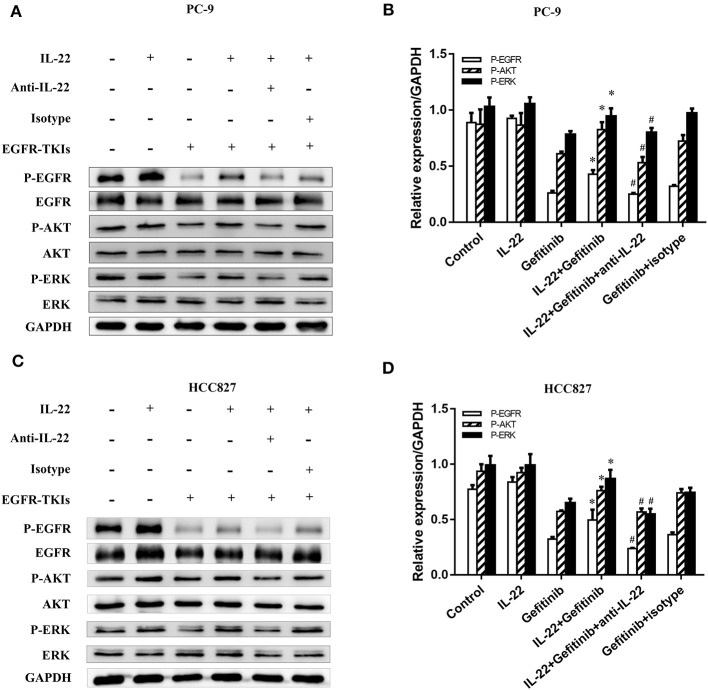
IL-22 promoted activation of the phosphorylated EGFR/AKT/ERK in NSCLC cells. **(A)** PC-9 and **(C)** HCC827 cells were incubated in the presence or absence of gefitinib (IC50) for 3 h prior to exposure to 50 ng/ml IL-22 (+/−), 10^4^ ng/mL IL-22 neutralizing antibody (+/−) and mouse IgG_1_ Isotype control (+/−) for additional 24 h, and the protein samples were analyzed by western blot with indicated antibodies. **(B)** PC-9 and **(D)** HCC827 cells densitometric analysis of western blots for the phosphorylated protein. ^*^P < 0.05 compared with gefitinib group. ^#^*P* < 0.05 compared with IL-22 plus gefitinib group. Data were presented as mean ± SEM of three independent experiments. anti-IL-22, IL-22 neutralizing antibody; Isotype, mouse IgG_1_ Isotype control.

PI3K/mTOR/AKT and MEK/ERK are two essential EGFR downstream signaling pathways (32). To detect the molecular mechanisms of acquired EGFR-TKI resistance, we confirmed that IL-22 affects crucial protein expression in these signaling pathways. We found a significant decrease in p-AKT, p-EGFR, and p-ERK expression in gefitinib-treated cells. IL-22 reversed the decreased expression of p-AKT, p-EGFR, and p-ERK caused by gefitinib. Moreover, IL-22 neutralizing antibody significantly abrogated the effect of IL-22 on gefitinib by down-regulating EGFR/AKT/ERK signaling (Figure 4). Together, these findings suggested that IL-22 mitigates damage caused by gefitinib, which is a factor in cellular resistance. The protective effect might be due to the up-regulation of p-AKT, p-EGFR, and p-ERK molecules.

### IL-22 Promoted Tumor Growth and Induced Gefitinib Resistance in the Xenograft Model of NSCLC

**Figure 5:**
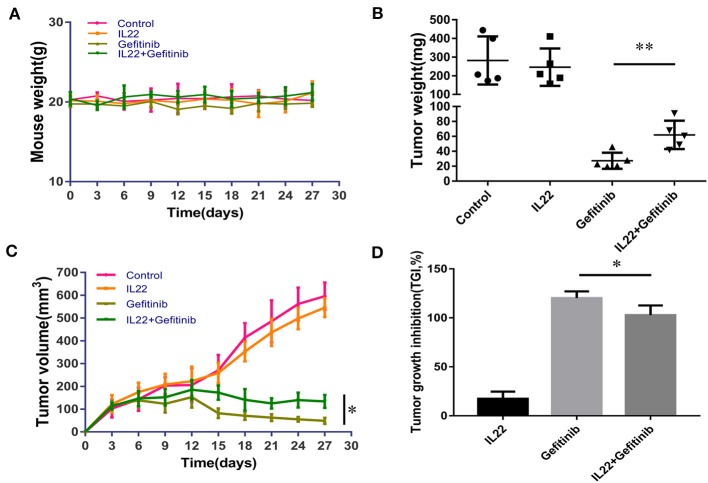
IL-22 induced resistance to gefitinib in the NSCLC xenograft model. **(A)** No substantial weight loss (≥20% of body weight at start of treatment) was observed in the mouse model during treatment. **(B,C)** Gefitinib significantly reduced the tumor volume and weight compared with control group. The addition of IL-22 to gefitinib treatment elevated tumor volume and weight compared with gefitinib alone (^*^P < 0.05, ^**^P < 0.01). Each group had five mice. **(D)** TGI (tumor growth inhibition) % by IL-22, gefitinib and gefitinib with IL-22 in the xenograft model on the last day of treatment (^*^P < 0.05).

Taking into consideration that IL-22 induced gefitinib resistance *in vitro*, we validated the IL-22 effects in the PC9 xenograft model. There was no substantial weight loss [≥20% of body weight at the start of treatment (33)] observed in the treatment (Figure 5A). IL-22 exerted no influence on tumor growth (volume, 546.53 mm^3^; weight, 246.26 mg) when compared with vehicle (volume, 596.28 mm^3^; weight, 281.96 mg, *P* > 0.05). Gefitinib significantly decreased tumor growth (volume, 48.75 mm^3^; weight, 27.3 mg) compared with vehicle treatment. Gefitinib treatment combined with IL-22 showed much lower antitumor activity (volume, 133.62 mm^3^; weight, 61.96 mg) compared with gefitinib alone [^*^P(volume) = 0.011; ^*^P(weight) = 0.008; Figures 5B,C]. Tumor growth inhibition (TGI %) for the gefitinib plus IL-22 group (102.9%) was less than the gefitinib group (120.2%; *P* value = 0.012; Figure 5D).

**Figure 6:**
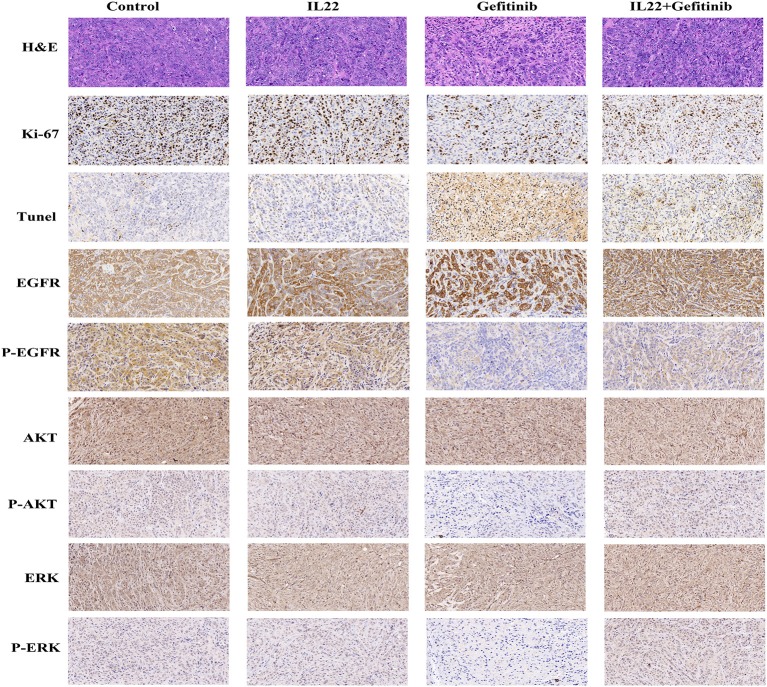
H&E, IHC, and TUNEL staining of tumor tissues. Tumor tissues of PC-9 xenografts (control, IL-22 alone, gefitinib alone, or gefitinib with IL-22) were processed and subjected to H&E, IHC, and TUNEL staining. The experiments were repeated at least 3 times, and a representative one is shown.

To confirm that IL-22 inhibited EGFR signaling pathways *in vivo*, we validated AKT, p-AKT, EGFR, p-EGFR, ERK, and p-ERK expression by IHC. It was shown that p-EGFR, p-AKT, and p-ERK expression was higher in the gefitinib plus IL-22-treated tumors and controls than the gefitinib monotherapy group (Figure 6). These *in vivo* data complemented the IL-22 *in vitro* findings, and further demonstrated that IL-22 may induce EGFR-TKI resistance *in vivo*.

The cytotoxic effect of gefitinib with or without IL-22 was further examined by Ki67 (a biomarker of tumor proliferation) and TUNEL staining analysis (evaluation of tumor apoptosis; Figure 6). Compared with gefitinib alone, the combination of IL-22 and gefitinib showed an increase in Ki67-positive staining and a reduction in TUNEL staining (both *P* < 0.05; Supplementary Table S1).

## Discussion

EGFR-TKIs have been widely used for the treatment of NSCLC patients harboring EGFR mutations (34). In the present study, we showed that IL-22 was correlated with EGFR-TKI acquired resistance in NSCLC harboring EGFR mutations.

Previous research has implicated that the immune system plays a role in both inherent and acquired resistance to targeted therapies (9). Mounting evidence suggests that a number of cytokines promotes cancer cell growth and mediates acquired resistance (35, 36). For example, CXCL-1/2 is associated with chemoresistance in lung cancer (37). A variety of immune cells have been reported to secrete IL-22 (22–24). To date, the role of IL-22 in tumors is unclear. IL-22 promotes colon cancer proliferation and stemness. IL-22 enhances pancreatic and gastric cancer invasion and migration. IL-22 also enhances the formation of liver cancer (38). However, other reports have shown that IL-22 inhibits proliferation and growth of tumor cells in a renal cell carcinoma line and a murine cell line of breast cancer (27). In lungs, IL-22 is expressed by macrophages, T cells, and alveolar epithelial cells (23, 39). IL-22 produced by immune cells and exogenous IL-22 prevents lung tissue from acute injury induced by viral infection or ventilation trauma (28, 40). Epithelial cells can react to IL-22 in lungs, where resident cell populations are able to produce IL-22. IL-22 is overexpressed in lung cancer tissues, malignant pleural effusions, and NSCLC patient serum (29). Also, IL-22 is elevated in lavage from patients with lung cancer (41). Compared to the primary tumor, recurrent NSCLC had higher levels of IL-22 expression (30). These evidences supply insight in the role of IL-22 for NSCLC patients.

IL-22 binds to the cell surface heterodimeric receptor complex that contains IL-10R2 and IL-22R1 to propagate downstream signals (27). IL-22 functions to regulate the inflammatory conditions of cancer (42), mediate cancer cell proliferation and migration, and modulate chemotherapeutic drug efficacy by altering the tumor microenvironment (30, 31). Recent studies have shown that IL-22 enhances proliferation and migration of lung cancer cells via the IL-22R1/AKT and IL-22R1/STAT3 signaling pathways (30). Also, IL-22 regulates apoptosis of paclitaxel-resistant NSCLC cells via the C-Jun N-terminal kinase signaling pathway (43). We focused on the function of IL-22 in EGFR-TKI resistance and found that IL-22 expression was higher in lung cancer tissues and plasma of patients with EGFR-TKI acquired resistance. We also showed that NSCLC cells demonstrated different sensitivity to gefitinib according to the IL-22 level in *in vitro* and *in vivo* studies. Because PI3K/mTOR/AKT and MEK/ERK are two essential EGFR downstream signaling pathways (30), we measured the p-AKT, p-EGFR, and p-ERK levels by western blot. IL-22 exposure significantly increased p-AKT, p-EGFR, and p-ERK expression after gefitinib treatment when compared with the group without IL-22 exposure. Similarly, previous studies have indicated that IL-8 confers resistance to EGFR-TKIs by increasing p-AKT (44), and IL-6 contributes to cisplatin resistance via activation of p-AKT and p-ERK in NSCLC (45).

Our results showed that the IL-22 targeting (IL-22 neutralizing antibody) strategy combined with conventional gefitinib therapy may reduce IL-22-induced gefitinib resistance. This has not been reported previously and has important clinical significance. No matter first-, second- or third-generation of EGFR-TKIs, drug resistance will eventually appear and induce to treatment failure. EGFR-TKIs combining with chemotherapy or antiangiogenic therapy are common strategy to delay disease progression. However, the side effects of these combination strategies also increase. The main strength of our study is providing new insight into clinical treatment for EGFR-TKI acquired resistance *in vitro* and *in vivo*. IL-22 targeting therapy will improve acquired gefitinib resistance and may have lower side effects. Also, it is convenient to dynamically monitor plasma IL-22 levels clinically. There are several limitations. Firstly, the sample size is small. Further larger samples are very needed to validate our results. Secondly, plasma IL-22 level may predict gefitinib resistance, but the threshold of plasma IL-22 or the ratio of IL-22 post-gefitinib and pre-gefitinib needs further study. Thirdly, third-generation of EGFR-TKI, osimertinib, is recently approved as the first-line standard in patients with EGFR mutation. The role of IL-22 in osimertinib treatment is an interesting issue.

Existing evidence indicates that resistance to EGFR-TKIs could be acquired through epigenetic and genetic changes in cancer cells, as well as tumor microenvironment activation. The NSCLC cells have been reported to produce endogenous IL-22 (29, 46). However, a study from Kobold et al. (31) showed that none of the cell lines (H187, H1339, LOU-NH91, HCC827, and A549) were found to express IL-22. We speculate that although IL-22 is also produced by lung cancer cells, the expression level may be very low. Expression of IL-22 in primary tissue may arise from non-tumor cells or from the interaction between tumor and non-tumor cells and stroma. Our findings showed that NSCLC cells with IL-22 exposure may acquire EGFR-TKI resistance, and have a better opportunity to survive compared with cells with lower IL-22 levels, suggesting that IL-22 paracrine signaling is indispensable. Considering the heterogeneity of tumors (there could be a mixture of IL-22-expression and non-IL-22-expression cells in tumors), a future investigation is needed to explore the endocrine function of IL-22 in triggering gefitinib resistance.

In sum, our study supplies compelling validation that IL-22 induces gefitinib resistance both *in vitro* and *in vivo*. IL-22-induced changes in the tumor microenvironment complicate EGFR-TKI acquired resistance, posing challenges for the clinical application of molecular targeted therapies.

## Data Availability Statement

The raw data supporting the conclusions of this manuscript will be made available by the authors, without undue reservation, to any qualified researcher.

## Ethics Statement

This study was carried out in accordance with the recommendations of institutional guidelines and Local Ethics Committee of the First Affiliated Hospital of Nanjing Medical University. All patients gave written informed consent in accordance with the Declaration of Helsinki. The protocols including animal experiment were approved by the Local Ethics Committee of the First Affiliated Hospital of Nanjing Medical University. The institutional guidelines of the Animal Care and Use of Nanjing Medical University were followed for the welfare of the animals.

## Author Contributions

WW and RG made substantial contributions to the design of the study. XW carried out the experiments and analyzed the data. JX and JC contributed to the data interpretation and drafting of the manuscript. SJ contributed to the review of previous literature. JY and TY contributed substantially to the data discussion and critically commented on the manuscript. RG and WW were responsible for the quality of the overall manuscript. All authors approved the final version of the manuscript.

### Conflict of Interest

The authors declare that the research was conducted in the absence of any commercial or financial relationships that could be construed as a potential conflict of interest.
