# Supplementary material for: IL-22 Confers EGFR-TKI Resistance in NSCLC via the AKT and ERK Signaling Pathways
Source: Front Oncol. 2019 Nov 5;9:1167. doi: 10.3389/fonc.2019.01167 (PMC6848259; doi:10.3389/fonc.2019.01167)
Supplement: Supplementary file 1 [file Table_1.DOCX]

**Supplementary Table S1** Quantification of Ki67 and TUNEL staining in different groups.

**Ki67**

| Group | n | Ki67(% positive cells) |
| --- | --- | --- |
| Control | 5 | 52.99 ±2.67 |
| IL-22 | 5 | 51.12 ±4.37 |
| Gefitinib | 5 | 33.96 ±3.84 |
| IL-22+ Gefitinib | 5 | 43.70 ±3.81*** |
| F | - | 26.976 |
| P | - | 0.001 |

**P*=0.001 vs Gefitinib group

**TUNEL**

| Group | n | Apoptosis (% positive cells) | |
| --- | --- | --- | --- |
| Control | 5 | | 12.89 ±2.13 |
| IL-22 | 5 | | 18.85 ±1.99 |
| Gefitinib | 5 | | 58.38 ±4.37 |
| IL-22+ Gefitinib | 5 | | 27.74 ±6.48*** |
| F | - | | 117.376 |
| P | - | | 0.000 |

**P* =0.000 vs Gefitinib group
